# Metastatic Squamous Cell Carcinoma to the Gallbladder Presenting as a Diagnostic and Surgical Challenge

**DOI:** 10.7759/cureus.98565

**Published:** 2025-12-06

**Authors:** Michael W Alchaer, Harrison Gorran, Amanda Rigdon, Thomas A Abbruzzese, Ji Fan

**Affiliations:** 1 General Surgery, HCA Healthcare/University of South Florida's (USF) Morsani College of Medicine GME/HCA Florida Brandon Hospital, Brandon, USA; 2 Internal Medicine, Lakeland Regional Health Medical Center, Lakeland, USA; 3 Gastrointestinal Surgery, Moffitt Cancer Center, Tampa, USA

**Keywords:** acute cholecystectomy, gallbladder metastasis, hepatobiliary surgery, robotic cholecystectomy, squamous cell carcinoma

## Abstract

Metastatic spread to the gallbladder is exceedingly rare, representing less than 5% of all gallbladder malignancies, with secondary involvement from head and neck squamous cell carcinoma (SCC) being exceptionally uncommon.

We present the case of a 68-year-old male with recurrent metastatic head and neck SCC who arrived with right upper-quadrant pain, nausea, and vomiting. Imaging demonstrated gallbladder distension and wall thickening without cholelithiasis, and subsequent hepatobiliary iminodiacetic acid (HIDA) scanning and magnetic resonance cholangiopancreatography (MRCP) findings supported a diagnosis of acute cholecystitis with cystic duct obstruction. The patient underwent robotic cholecystectomy, during which indocyanine green (ICG) fluorescence cholangiography was utilized to delineate biliary anatomy in the setting of severe inflammation. Intraoperative evaluation revealed a gangrenous gallbladder with cystic duct necrosis. Histopathologic examination demonstrated a 1.5 cm epithelioid/squamoid carcinoma consistent with metastatic SCC, with positive cystic duct margins. The postoperative course was uneventful.

Metastatic SCC to the gallbladder is often indistinguishable from benign inflammatory disease on imaging and is commonly diagnosed only after cholecystectomy. While prognosis is largely determined by the burden of systemic disease, surgical excision of isolated lesions may provide symptomatic relief and facilitate diagnosis. This case highlights the importance of considering metastatic disease in patients with a history of malignancy who present with cholecystitis-like symptoms and underscores the value of minimally invasive cholecystectomy in achieving safe management and diagnostic clarity.

## Introduction

Metastatic tumors of the gallbladder are exceedingly rare, accounting for less than 5% of all gallbladder malignancies [1,2]. Common primary sites include the stomach, pancreas, and lung, whereas metastases from squamous cell carcinoma (SCC) of the head and neck are exceptionally uncommon [3,4]. The gallbladder’s mucosal barrier and limited lymphatic drainage are thought to confer resistance to metastatic implantation [5,6].

When metastasis occurs, it usually results from hematogenous or lymphatic spread rather than direct invasion [7]. These lesions often present with clinical and radiologic findings indistinguishable from acute or chronic cholecystitis, making preoperative diagnosis challenging [7,8]. Robotic cholecystectomy has emerged as a valuable tool for complex biliary pathology due to its superior visualization and precision [9,10]. We present a unique case of metastatic SCC to the gallbladder masquerading as acute cholecystitis, managed successfully with robotic cholecystectomy and indocyanine green (ICG) cholangiography.

## Case presentation

A 68-year-old male presented to the hospital with right upper quadrant abdominal pain, accompanied by nausea and vomiting that had been worsening over two days, particularly after meals. His medical history was significant, including recurrent metastatic squamous cell carcinoma of the head and neck for which he had undergone multiple rounds of neck radiation, surgical resection, chemotherapy, and immunotherapy. Additionally, he had a history of atrial fibrillation, follicular lymphoma, and chronic obstructive pulmonary disease (COPD) requiring 3 L/min nasal-cannula oxygen at home.

**Figure 1:**
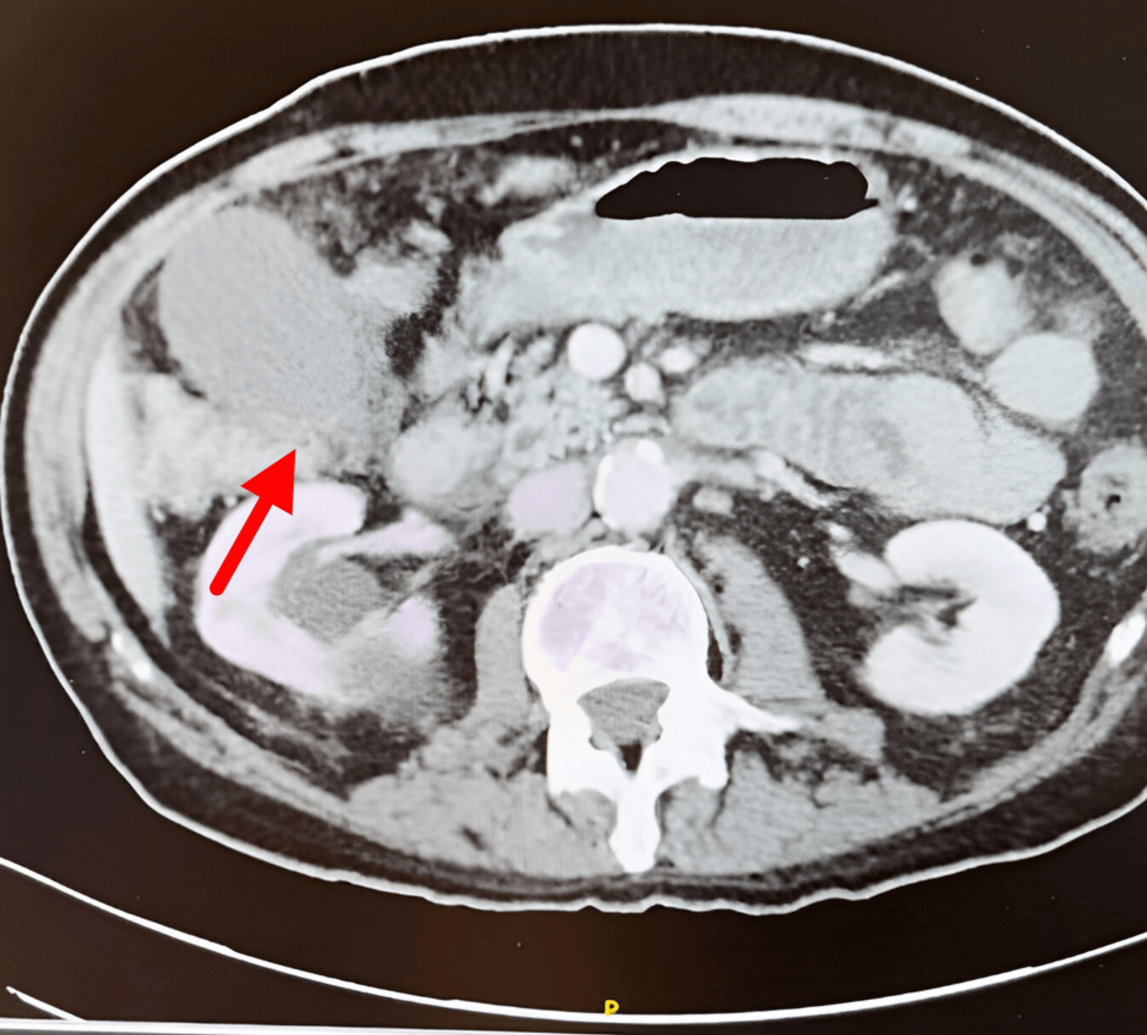
Coronal Slices of CT Thoracic Abdomen and Pelvis without contrast: Gallbladder Distension Arrow pointing at distended gallbladder with wall thickening.

Initial workup revealed anemia, with a total bilirubin of 0.4 mg/dL and Aspartate Aminotransferase/Alanine Aminotransferase (AST/ALT) within normal limits (Table 1). A right upper quadrant ultrasound showed gallbladder wall thickening without evidence of cholecystitis or cholelithiasis. A CT scan of the abdomen and pelvis with contrast demonstrated increased gallbladder distention and wall thickening (Figure 1). A subsequent hepatobiliary iminodiacetic acid (HIDA) scan showed non-visualization of the gallbladder despite the morphine challenge, consistent with acute cholecystitis and cystic duct obstruction. Magnetic resonance cholangiopancreatography (MRCP) findings included acute cholecystitis, mild intrahepatic ductal dilation with peribiliary edema suggestive of cholangitis, and no discrete stones or choledocholithiasis, raising concern for possible cystic duct stenosis.

**Table 1:** Reference Laboratory Values

Test	Patient Value	Reference Range	Units
Temperature	98.4	97.8 to 99.1	degrees Fahrenheit
Heart rate	92	60 to 100	beats per minute
Systolic blood pressure	134	90 to 120	millimeters of mercury
Diastolic blood pressure	78	60 to 80	millimeters of mercury
Respiratory rate	18	12 to 20	breaths per minute
Oxygen saturation	95	95 to 100	percent
Hemoglobin	10.2	13.5 to 17.5	grams per deciliter
Hematocrit	31	41 to 53	percent
White blood cell count	9.8	4.0 to 11.0	times ten to the third per microliter
Platelets	210	150 to 400	times ten to the third per microliter
Total bilirubin	0.4	0.2 to 1.2	milligrams per deciliter
Aspartate aminotransferase	22	10 to 40	units per liter
Alanine aminotransferase	24	7 to 56	units per liter
Alkaline phosphatase	88	44 to 147	units per liter
Creatinine	0.9	0.7 to 1.3	milligrams per deciliter
Blood urea nitrogen	14	7 to 20	milligrams per deciliter

Based on these findings, the patient was scheduled for robotic cholecystectomy. The intraoperative scenario proved to be exceptionally challenging. Upon exploration, we found the gallbladder and cystic duct to be grossly gangrenous. We utilized Indocyanine Green (ICG) cholangiography to identify the biliary anatomy intraoperatively, which proved invaluable given the distorted tissue planes. Interestingly, the distal cystic duct, once dissected free, appeared non-necrotic.

**Figure 2:**
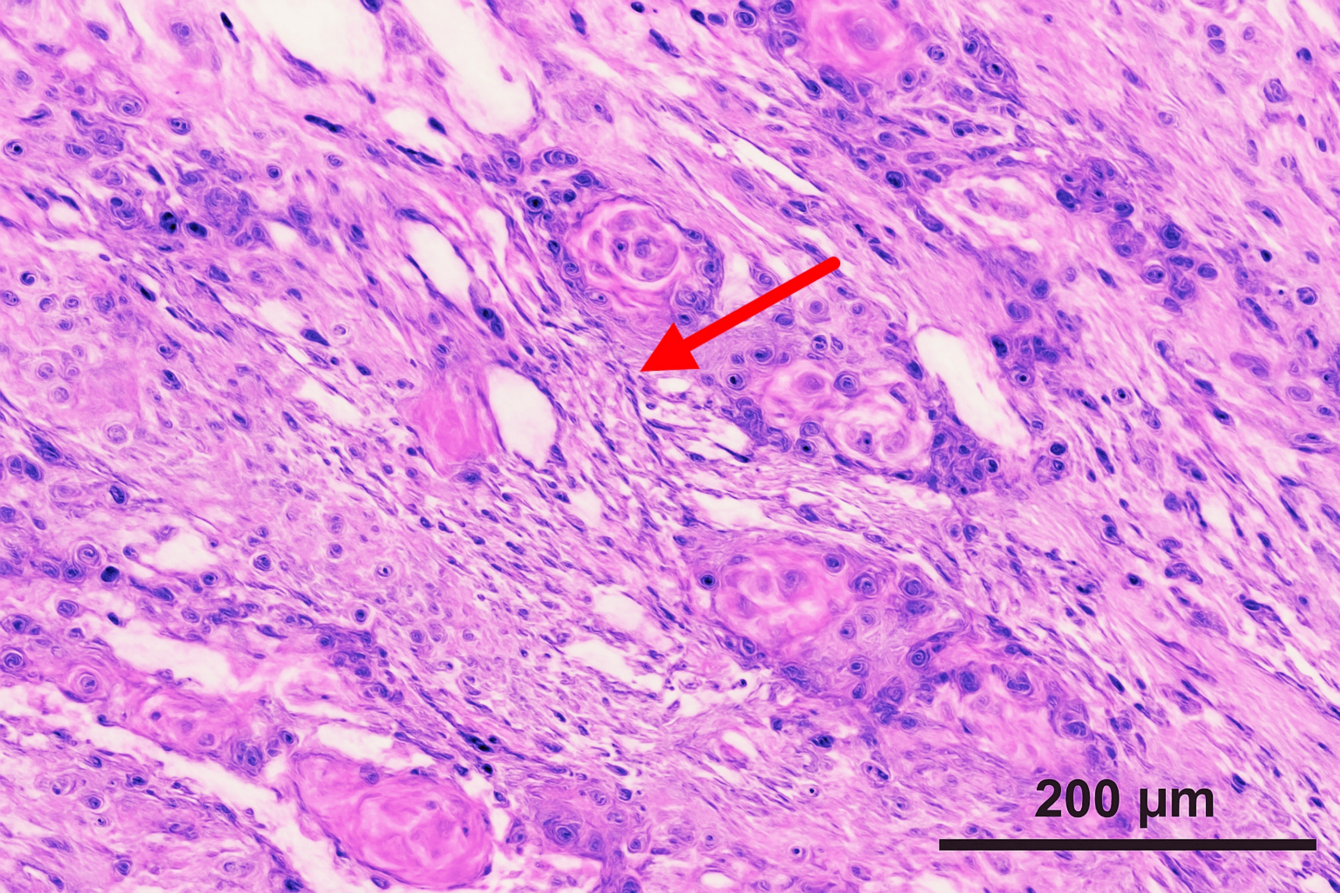
Histology of Squamous Cells in the Gallbladder Magnification 200x, arrow pointing at squamous cells in the gallbladder.

The pathology report following cholecystectomy revealed a surprising finding: the gallbladder contained a malignant epithelioid/squamoid tumor measuring 1.5 cm, accompanied by gangrenous cholecystitis (Figure 2). Moreover, the cystic duct margins were positive for malignancy. Despite the complexity of the case, the patient's postoperative course was uncomplicated.

## Discussion

Metastatic involvement of the gallbladder is rare, with autopsy series estimating an incidence of approximately 0.5 to 3 percent among cancer patients [1,5]. The most frequently reported primary malignancies include melanoma, renal cell carcinoma, and breast cancer [11-13]. When metastases occur, they generally result from hematogenous or lymphatic dissemination rather than direct contiguous spread [6,7,14,15].

Visceral metastasis from head and neck squamous cell carcinoma (SCC) is uncommon and most commonly affects the lungs or bones. Gallbladder involvement is exceedingly rare and typically arises in the setting of disseminated disease [3,4,16]. The continuous flow of bile and the gallbladder mucosal barrier are thought to limit tumor implantation. However, mucosal injury or localized ischemia may facilitate metastatic seeding [5,7].

Radiologic findings of metastatic gallbladder disease are often nonspecific and frequently mimic benign inflammatory conditions such as acute or chronic cholecystitis [7,8]. Ultrasound and computed tomography (CT) commonly demonstrate gallbladder wall thickening or intraluminal masses, but these findings are insufficient to reliably differentiate malignancy from inflammation [7,8]. In the present case, preoperative imaging, including MRCP and HIDA scanning, suggested acute cholecystitis without visualization of a mass, highlighting the diagnostic limitations of current imaging modalities. Positron emission tomography combined with computed tomography (PET-CT) may improve detection in patients with known malignancy but remains limited by modest specificity [5]. Definitive diagnosis, therefore, relies on histopathologic evaluation following cholecystectomy [7,8].

Inflammatory distortion, neoplastic infiltration, or both can obscure biliary anatomy and increase the risk of bile duct injury during cholecystectomy [10,17]. Robotic platforms may reduce this risk by providing enhanced visualization, depth perception, and instrument dexterity during complex dissections, especially when combined with ICG fluorescence cholangiography [6,9].

Published literature indicates that metastatic gallbladder involvement, including metastases from SCC and other primary tumors, is frequently identified incidentally after cholecystectomy performed for presumed benign disease [1,7,8]. Yoon et al. reported multiple cases in which all metastases were misdiagnosed preoperatively [18]. Similarly, Cocco et al. emphasized the importance of maintaining a high index of suspicion in patients with a history of malignancy presenting with cholecystitis-like symptoms [8].

Prognosis is driven predominantly by the extent of systemic disease rather than the gallbladder lesion itself [1,2,4]. In rare cases of isolated gallbladder metastasis, surgical excision may provide symptomatic relief and diagnostic clarity, although systemic therapy remains the cornerstone of treatment [2-4,14]. Given its rarity and nonspecific presentation, careful interpretation of imaging studies and heightened intraoperative vigilance are essential to avoid delays in diagnosis and ensure safe operative management [6,9,10,17].

## Conclusions

Gallbladder metastases from head and neck squamous cell carcinoma are exceptionally uncommon and pose a significant diagnostic challenge because they closely resemble benign inflammatory disease on imaging. Recognition of this possibility is essential in patients with a history of malignancy who present with symptoms similar to acute cholecystitis. Histopathologic evaluation following cholecystectomy remains the definitive method of diagnosis. While prognosis depends primarily on the extent of systemic disease, surgical excision of isolated lesions can provide both diagnostic clarity and symptomatic relief. Clinicians should maintain a high index of suspicion for metastatic involvement in oncology patients presented with new atypical right upper quadrant pain, even when imaging suggests acute cholecystitis, as metastatic disease often mimics benign inflammation. Given that histopathology is the only definitive diagnostic tool, timely cholecystectomy and careful intraoperative assessment are crucial to avoid delays in diagnosis and to guide appropriate oncologic management.
